# Public Deliberation Process on Patient Perspectives on Health Information Sharing: Evaluative Descriptive Study

**DOI:** 10.2196/37793

**Published:** 2022-09-16

**Authors:** Minakshi Raj, Kerry Ryan, Paige Nong, Karen Calhoun, M Grace Trinidad, Raymond De Vries, Melissa Creary, Kayte Spector-Bagdady, Sharon L R Kardia, Jodyn Platt

**Affiliations:** 1 Department of Kinesiology and Community Health University of Illinois at Urbana Champaign Champaign, IL United States; 2 Center for Bioethics and Social Sciences in Medicine University of Michigan Ann Arbor, MI United States; 3 School of Public Health University of Michigan Ann Arbor, MI United States; 4 Michigan Institute for Clinical & Health Research Ann Arbor, MI United States; 5 National Hemophilia Program Coordinating Center Ann Arbor, MI United States; 6 Department of Learning Health Sciences University of Michigan Ann Arbor, MI United States; 7 Department of Obstetrics and Gynecology University of Michigan Ann Arbor, MI United States

**Keywords:** public deliberation, data sharing, health information exchange, patient engagement, health information, sharing, cancer, oncology, precision oncology, information, policy, personalized medicine, public preference

## Abstract

**Background:**

Precision oncology is one of the fastest-developing domains of personalized medicine and is one of many data-intensive fields. Policy for health information sharing that is informed by patient perspectives can help organizations align practice with patient preferences and expectations, but many patients are largely unaware of the complexities of how and why clinical health information is shared.

**Objective:**

This paper evaluates the process of public deliberation as an approach to understanding the values and preferences of current and former patients with cancer regarding the use and sharing of health information collected in the context of precision oncology.

**Methods:**

We conducted public deliberations with patients who had a current or former cancer diagnosis. A total of 61 participants attended 1 of 2 deliberative sessions (session 1, n=28; session 2, n=33). Study team experts led two educational plenary sessions, and trained study team members then facilitated discussions with small groups of participants. Participants completed pre- and postdeliberation surveys measuring knowledge, attitudes, and beliefs about precision oncology and data sharing. Following informational sessions, participants discussed, ranked, and deliberated two policy-related scenarios in small groups and in a plenary session. In the analysis, we evaluate our process of developing the deliberative sessions, the knowledge gained by participants during the process, and the extent to which participants reasoned with complex information to identify policy preferences.

**Results:**

The deliberation process was rated highly by participants. Participants felt they were listened to by their group facilitator, that their opinions were respected by their group, and that the process that led to the group’s decision was fair. Participants demonstrated improved knowledge of health data sharing policies between pre- and postdeliberation surveys, especially regarding the roles of physicians and health departments in health information sharing. Qualitative analysis of reasoning revealed that participants recognized complexity, made compromises, and engaged with trade-offs, considering both individual and societal perspectives related to health data sharing.

**Conclusions:**

The deliberative approach can be valuable for soliciting the input of informed patients on complex issues such as health information sharing policy. Participants in our two public deliberations demonstrated that giving patients information about a complex topic like health data sharing and the opportunity to reason with others and discuss the information can help garner important insights into policy preferences and concerns. Data on public preferences, along with the rationale for information sharing, can help inform policy-making processes. Increasing transparency and patient engagement is critical to ensuring that data-driven health care respects patient autonomy and honors patient values and expectations.

## Introduction

### Current State of Precision Oncology

Precision oncology is one of the fastest-developing domains of personalized medicine [1-5]. Genomic testing and molecular profiling of tumors that indicate highly targeted therapies are increasingly available in routine medical practice. Delivery of this type of care is a highly data-intensive enterprise, requiring the processing of electronic health records (EHRs), genomic sequence data, and patient-reported outcomes, among other types of data, from entire patient populations without patients’ knowledge. Current policies, such as the 21st Century Cures Act, incentivize interoperability of data and stand to accelerate personalized medicine and other data-driven enterprises, such as learning health systems and artificial intelligence (AI)-enabled clinical decision support. Greater interoperability is a policy goal in order to enable data exchange for use by health systems, commercial companies, laboratories capable of performing genome sequencing, and registries that facilitate disease surveillance and monitoring. However, the policies governing the data ecosystem for precision health and related enterprises are typically opaque to patients, particularly when data are collected in the context of clinical care [6-9]. This paper evaluates the process of public deliberation as an approach to understanding the values and preferences of current and former cancer patients for different notification strategies that may be used to increase transparency about how health information is used and shared.

A number of strategies have been proposed for notifying people about how their data are used in the context of research data and biospecimens (eg, biobanks), which could be extended to the context of sharing clinical data. For instance, there has long been an emphasis on educating the public about health information sharing in “plain language,” that is, using vernacular that is accessible to readers. This type of notification about the uses of health information could be delivered as signs posted in hospitals, clinics, or doctor’s offices [10]. In the context of biobanking and longitudinal cohort studies, previous research has indicated public preferences for more notification each time health information is used or shared [11]. Technology such as patient portals or messaging via email or text could be leveraged to notify patients when, by whom, and for what purpose their clinical data are shared [12]. Previous work has also suggested that notification will be insufficient and argues that the ability to exercise autonomy and the ability to opt out of certain data uses is ethically required [13]. Given the commercial aspects of data use, still others have suggested payment for the use of personal data, which could extend to health data [14]. These different options to notify and maintain transparency with patients about clinical data sharing highlight different potential roles and responsibilities for the public in the health information ecosystem. What is more, patient preferences about these different approaches to notification remain unclear.

### Deliberation to Understand Public Preferences

Here, we describe our use of a public deliberation approach to understand data sharing preferences of current and former cancer patients, particularly related to the use and sharing of clinical data. Public deliberation is a process that facilitates public input on social issues to develop policies and identify issues for future research that reflect public preferences [15]. The deliberative approach affords several benefits compared to interviews, focus groups, and surveys. For example, deliberations often bring together people with diverse values, opinions, interests, and life experiences, as well as diverse socioeconomic (eg, income and education) and racial backgrounds, offering an ideal opportunity for identifying commonalities in policy preferences across diverse deliberators [16]. They also reveal key complexities in decision-making processes and outcomes, enabling the generation of new policy recommendations with a better understanding of public preferences and the values underlying those preferences [17-19]. Public deliberation provides an opportunity to share information with participants (ie, deliberators) and to solicit perspectives at multiple levels (ie, from individuals, small groups, and the collective of deliberators). Since deliberators provide insights about real-life scenarios and voice their preferences, the deliberation process can also be empowering, giving deliberators the opportunity to actively develop and shape policies rather than simply being impacted by them [20].

### Methods of a Deliberative Approach

We used a framework for describing public deliberation methods articulated by De Vries and colleagues [17,21] to guide our analysis. The framework proposes that 3 dimensions—*process, information,* and *reasoning*—reflect key characteristics of the deliberative approach and capture the primary methods that comprise deliberations. The *process* dimension is concerned with the design and implementation of the project itself. The *information* dimension considers whether and to what extent participants apply information presented to them in their discussions and seek new information. The *reasoning* dimension considers how participants balance and navigate different perspectives and how they ultimately reach mutual understanding within their group about a policy. In the current study, we sought to understand the perspectives of current or former cancer patients on the use and sharing of health information and on potential organizational policy that might increase transparency. Here, we describe the methodology of a public deliberation along the dimensions of *process*, *information*, and *reasoning* to inform investigation of other issues in the development and implementation of health information policy that would benefit from public input.

## Methods

We conducted two public deliberations in the fall of 2019 with current or former cancer patients in Southeastern Michigan to hear their perspectives on how health information should be used, shared, and regulated.

### Ethical Considerations

This study was approved by the University of Michigan Institutional Review Board and was deemed exempt from federal regulations (HUM 00158768). Participants provided written consent prior to participation.

### Participant Recruitment

We recruited former and current cancer patients through a research platform managed by a large Midwestern academic health center [22]. The platform, resourced by the university’s Clinical and Translational Science Institute, has a pool of nearly 48,000 individuals who represent a partnership between researchers and volunteers to encourage participant recruitment in research. Eligibility criteria for this study included comfort with speaking English, age 21 years or older, and a former or current diagnosis of cancer of any type. The study team also recruited purposively to ensure diversity in race, age, education, and gender. A total of 79 participants were enrolled, of whom 61 attended either session 1 (n=28) or session 2 (n=33). Table 1 summarizes the demographic characteristics of the participants. To recognize the participants’ full-day contribution, they received US $100 and meals (breakfast and lunch).

**Table 1:** Demographic characteristics of participants (N=61).

Characteristics			Values
**Gender, n (%)**			
	Female	36 (59%)	
	Male	25 (41%)	
Age (years), mean (SD)			62.1 (10.2)
**Race/ethnicity^a^, n (%)**			
	African American or Black	11 (18%)	
	American Indian or Alaska Native	2 (3%)	
	Asian American or Asian	2 (3%)	
	Hispanic or Latino	3 (5%)	
	Middle Eastern or Arab American	0 (0%)	
	Pacific Islander or Hawaiian Native	0 (0%)	
	White	44 (72%)	
	Other	1 (2%)	
**Highest level of school completed, n (%)**			
	Less than Bachelor of Arts	16 (26%)	
	Bachelor of Arts	20 (33%)	
	More than Bachelor of Arts	25 (41%)	
Working in health care field, n (%)			16 (26%)
**Household income, n (%)**			
	Less than $50,000	23 (38%)	
	$50,000 to $75,000	9 (15%)	
	$75,000 to $100,000	9 (15%)	
	$100,000 to $150,000	9 (15%)	
	More than $150,000	5 (8%)	
	Prefer not to answer	6 (10%)	
**Employment status, n (%)**			
	Working	21 (34%)	
	Not working (retired)	24 (39%)	
	Not working (disabled)	11 (18%)	
	Not working (other)	4 (7%)	
	Prefer not to answer	1 (2%)	
**Health status^b^, n (%)**			
	Excellent	7 (12%)	
	Very good	21 (35%)	
	Good	21 (35%)	
	Fair	10 (17%)	
	Poor	1 (2%)	

^a^Participants were allowed to select more than one response.

^b^The total is less than 61 due to missing information from 1 participant.

### Deliberation Process

**Figure 1:**
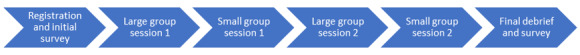
Components of deliberation sessions.

For small group discussions, participants were randomly assigned to 1 of 5 groups with 6 to 8 participants in a large meeting space. Each small group had a facilitator trained in deliberative engagement principles. Eligible participants received an educational booklet that included a description of the study and overview of key terms by mail prior to the session. The educational booklet is included in Multimedia Appendix 1. Figure 1 summarizes the primary components of the deliberation sessions; the processes were identical for both sessions.

At the beginning of the session, participants completed a 20-minute survey about their views on health data sharing. Study team experts led one plenary session in the morning with the full group to provide information about how and why health data are collected, stored, and shared, along with major ethical considerations associated with health data sharing. Fifteen minutes were dedicated at the end of the initial informational session for participants to ask questions. This was followed by small group discussions about a scenario reflecting the life cycle of health data and policy preferences described in the plenary session (scenario A). After lunch, the same process was used with a second plenary presentation about the role of commercial companies in precision oncology and commercialization of health information. This was followed by questions and answers and small group discussions on a scenario and set of options for governing data sharing with commercial companies (scenario B). Both scenarios are described below. The second small group was followed by a final large group session, in which the small group facilitators reported their groups’ preferences to the large group as a whole. At the end of the session, participants completed another 20-minute survey on health data sharing. The session agenda is available in Multimedia Appendix 2. A description of the two scenarios follows.

#### Scenario A: General Policy Preferences Related to Health Data Sharing

Participants read a 1-page scenario describing a patient with early-stage breast cancer whose information is added to a hospital cancer registry. Through the state health information exchange, the patient’s information is shared between her health care providers and between hospital, state, and national registries that collect information about cancer over time, and also prompts her provider when it is time for a checkup. Participants were given a summary of the current policy related to this kind of health information sharing and then asked to consider the 4 scenario A policy options in Textbox 1.

In small groups, participants independently ranked their individual preference for each policy option from most preferred to least preferred. Individual responses were then aggregated to form a group-level ranking. Participants shared their concerns and considerations by framing their preferences in a facilitated discussion to arrive at a group-level recommended prioritization of options. At the end of the discussion, the facilitator asked participants to consider the policies once again to assess whether their individual preferences changed during the discussion. The final rankings for each small group were then aggregated with all the other groups in the session to arrive at a final session-level set of policy preferences.

**Policy options for deliberative dialogue sessions.**

Scenario A. Preferences for notification about data sharing

1. “Plain language”—signs posted in clinics and hospitals
2. Text or email notification
3. Data sharing policy and instances displayed in patient portals
4. No change from current policy

Scenario B. Preferences for notification and policies for use of information by commercial companies

1. Data sharing policy and instances displayed in patient portals
2. Text or email notification
3. Opt-out of sharing data with commercial companies
4. Compensation—receive payment when data are accessed or used
5. No change from current policy

#### Scenario B: Policy Preferences on Health Data Sharing With Commercial Companies

We used the same process for scenario B in the afternoon session. This scenario described the same patient with early-stage breast cancer whose doctor suggests she undergo genetic testing to identify a tailored treatment. The doctor sends samples of her tumorous and healthy cells to a commercial company for genetic testing without her knowledge. Although the company sends results back to her doctor, the company retains her samples due to an agreement with the hospital to continue testing and aggregating samples from thousands of patients to ultimately advance research and treatments. The company can also sell samples to other companies, generating revenue from sales of samples and patient data. Participants were then given a summary of the current policy related to health information sharing and asked to consider 5 policy options, which they ranked in order of preference. The 5 scenario B policy options are summarized in Textbox 1.

### Analysis

Audio recordings of the small group discussions were transcribed verbatim and deidentified. The framework for public deliberation described by De Vries and colleagues [21], which guided our descriptive study, includes 3 dimensions—*process*, *information*, and *reasoning—*which reflect key components of the deliberative approach that capture its key characteristics.

We describe the process of deliberations by considering the design and implementation of the project, including facilitation style, participant engagement, and respectful group dynamics. In our results, we also describe the preparation process for deliberations and descriptively analyze responses to survey items that assessed the quality of some aspects of the deliberation process methods. The following survey questions were answered on a Likert scale ranging from 1 (“not at all”) to 10 (“very much”): “Do you feel you were listened to by your facilitator?” “Do you feel your opinions were respected by your group?” and “Do you feel the process that led to your group’s responses was fair?”

The *information* dimension of the framework captures the extent to which participants apply information presented in educational sessions in their discussions and seek new information to make sense of complex issues. This dimension reflects whether participants use on-site experts, integrate new information, and apply this new information to form policy opinions. We analyzed the *information* dimension of our approach through qualitative and quantitative analyses: we (1) developed a qualitative code reflecting instances when participants recalled or reflected on something they learned in the educational session in their discussion; (2) assessed when a group would seek additional information from on-site experts; and (3) quantitatively analyzed whether participants learned new information by using the McNemar test to compare each participant’s pre-and postdeliberation responses to a series of true or false prompts about health information sharing. These prompts were a part of a postdeliberation survey (Multimedia Appendix 3) that included the following statements: (1) “Current health privacy laws prevent private companies from buying or accessing your health information” (false); (2) “State and local health departments collect information from physicians and clinics to monitor health” (true); (3) “Only health care providers can access medical records” (false); and (4) “Your physician determines all uses of information in your medical record” (false).

The *reasoning* dimension of the framework reflects whether and how participants navigate different points of view and reach consensus or mutual understanding about their position on a policy. This dimension assesses participants’ ability to justify their opinion with reasoning, their openness to complexity, and their adoption of a societal perspective (ie, thinking beyond their individual self-interest). We qualitatively coded transcriptions for expressions of the pros and cons of the various policy options, including the rationales deliberators provided for their positions, instances in the discussions where they presented multiple perspectives on an issue, and discussions related to the benefits and risks of different policy options to society.

## Results

### Deliberator Characteristics

The mean age of the participants (N=61) was 62.1 (SD 10.2) years, 72% (44/61) were non-Hispanic white, and 59% (36/61) identified as female. Nearly three-quarters (45/61, 74%) of participants had at least a bachelor’s degree and 26% (16/61) worked in health care.

#### Process

Examining the *process* dimension entailed focus on 3 areas: facilitation, participant engagement, and respectful group dynamics. Facilitators were given training materials summarizing the purpose and goals of the deliberative approach, prompts for small group discussions, and best practices for facilitation (eg, “fading into the background,” encouraging discussion between participants rather than through the facilitator, ensuring all participants had an opportunity to contribute, and utilizing conflict resolution strategies). Facilitators also underwent a 2-hour training session prior to the deliberations to orient them on all the materials. They also observed how a previously trained facilitator led a “mock” discussion and asked questions about the process (eg, policy ranking) and best practices (eg, active listening and ensuring inclusivity in small group discussions).

We captured participants’ perceptions of facilitation and respectful group dynamics through a survey with a Likert scale ranging from 1 (not at all) to 10 (very much). Participants on average felt they were listened to by their facilitator (mean score 9.9, SD 0.3), their opinions were respected by their group (mean score 9.7, SD 0.8), and that the process that led to their group’s responses was fair (mean score 9.8, SD 0.6).

#### Information

**Figure 2:**
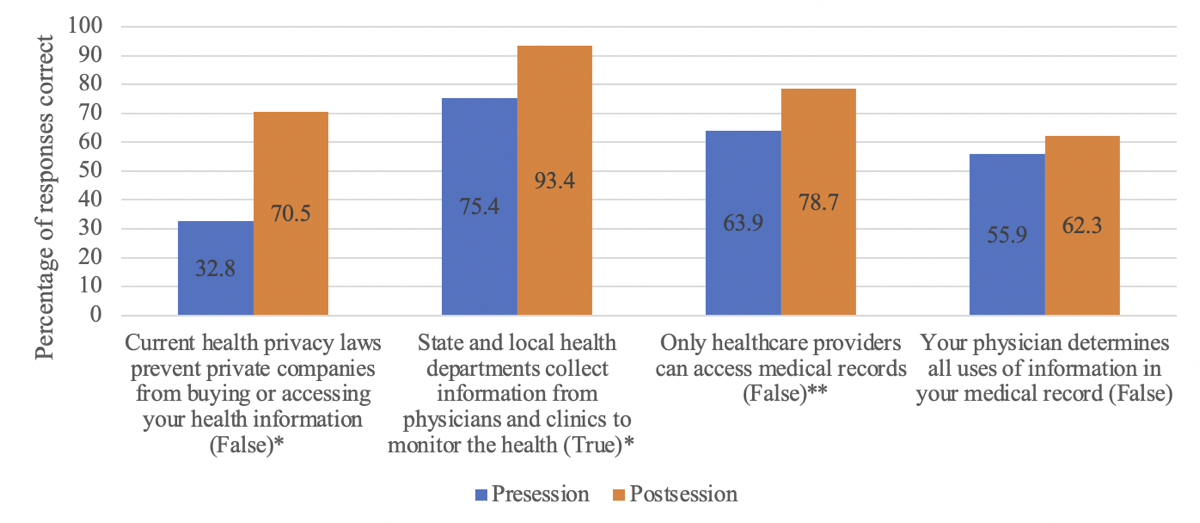
Changes in deliberators' knowledge pre- and postsession (N=61). *P<.01, **P<.1.

In our approach, we initially prepared the *information* dimension as educational materials and presentations, designated time for questions and answers, and then encouraged participants to use on-site experts as needed during small group discussions. We used postdeliberation survey responses to assess whether participants learned new information and formed opinions based on new information (Figure 2). The following informational components were reported to be very or extremely helpful (at least 8 on a 10-point scale) by at least 90% of respondents: (1) questions and answers with experts (56/61, 92%); (2) formal presentations given by the experts (57/61, 94%); and (3) discussing the issues with other participants (57/61, 94%). In addition, most participants reported that attending the session changed both their understanding of health information sharing (46/61, 77%) and their opinions about health information sharing (36/60, 60%). Finally, analysis of responses from the 4 knowledge questions indicated that participants gained knowledge throughout the course of the session, with the McNemar test indicating significant differences in prompts about current health privacy laws preventing private companies from buying or accessing health information (*P*<.001) and prompts about state and local health departments collecting information from physicians and clinics to monitor health (*P*=.003).

Qualitative analysis also revealed that participants were integrating new information from the educational sessions into their small group discussions. For example, one participant drew on the educational presentations to talk about the role of trust and what it meant in relation to data sharing:

One of the things that was mentioned a couple times in the presentation is the word “trust.” Two different medical entities having different types of information. You almost have to either trust them or not trust their honesty and integrity as to how they’re going to use that.

Deliberation #2, group 1

#### Reasoning

Qualitative analysis of deliberations indicated that participants demonstrated reasoning when engaging in discussions about policy preferences during the process of reaching consensus. They engaged with trade-offs about both individual and societal perspectives, recognized complexity, and made compromises related to health data sharing. For example, participants supported the patient portal policy because they would have the flexibility of accessing the information at their convenience without the overwhelming nature of receiving notifications via text message. One participant explained their individual-level perspective:

I picked [access through patient portal] for my first choice because by the time I had my cancer treatments, I was quite ill, and I was not able to really speak for myself or understand things that were being thrown at me to sign. So I like the idea where you can wait until you’re able to think more clearly.

Deliberation #2, group 3

At the same time, participants also looked beyond themselves, and recognized societal barriers to patient portal access:

So I don’t think that the portal...or the smartphone...is really adequate to address the needs of certain communities.... There may be barriers in terms of getting to the locations that have the computers...just being able to get there transportation wise, but perhaps also work-life schedules not lining up with those public institutions and things. So those would be barriers.

Deliberation #1, group 2

They had similar individual concerns about notifications:

“My husband wouldn’t know what to do if he got a text on his phone. You know, he’s got a smartphone, but he [only] makes calls on it.”

Deliberation #2, group 2

However, they also demonstrated consideration of a societal perspective:

The problem I have is that not everybody has a cell phone. Not everybody has access to electronics, and probably the people who are most underserved are those people. Probably the socioeconomic group odds are they don’t have money to buy these fun things, or they don’t have the education to be able to use them. So they’re left in the dark, and they’re probably the ones that are most easily taken advantage of.

Deliberation #1, group 1

Respondents recognized the complexity of different policy options and expressed concerns regarding the sufficiency of information through plain language communication, such as feeling overwhelmed by dense documentation with difficult jargon. When asked to explain the pros and cons of individual policy options to reach consensus, participants agreed nearly unanimously that the current policy was not working well:

Well, I did not know how freely they could share the information, that they are actually sharing them with payers. So, something needs to be done with that because we have a right to know where our information is going.... I sure wouldn’t want it to start impacting hiring practices, even issues...reproductive rights, your insurance, your housing, all that.... I never thought about that when I signed up for All of Us, but now that it’s out there...that concerns me because it can be used against us...and discrimination can occur.

Deliberation #1, group 1

Participants also suggested modifications to policies, such as being notified and then having the opportunity to access the portal for further information. These suggestions reflected that participants were making compromises to enable health data sharing while maintaining their comfort boundaries:

I chose to receive a text or email when my information is shared, but I would like that tweaked a little bit. I would like...just like they ask, “Is it okay to send the email, is it okay to access your Google account, is it okay to change this or change that,” and you say, “Okay.” I would like them to say, “is this okay?” And then you can answer it. You have a choice in whether they share it with whoever they’re sharing it with.

Deliberation #2, group 1

## Discussion

### Principal Findings

Precision oncology is a data-intensive medical field involving multiple stakeholders. This study used a public deliberation approach to seek patient input on whether and how they would like greater transparency about the data sharing that is necessary to deliver precision care. Given the complexity of the field, a deliberative methodology best fit our goal of understanding preferences and the rationale behind those preferences. In our deliberations, the *process* dimension of conducting a deliberation involved training facilitators and establishing rapport among small groups of deliberators. We provided the *information* dimension to deliberators through educational sessions, and nearly all participants reported they found the information helpful and that it enhanced their understanding of health data sharing. Deliberators also integrated information into their small group discussions, using it to form opinions and navigate complexities of policies and the risks and benefits associated with each. Through this *reasoning* dimension, they ultimately reached mutual understanding about different policy options.

The deliberative process fostered an environment in which participants could collaboratively suggest modifications to policies and reach mutual understanding about the policies, as well as a broad range of considerations that guided their opinions. We found participants reasoned with complexities related to the practical, ethical, and social implications of health data sharing. For instance, participants noted the barriers to using the patient portal to share information, particularly in an environment with a persistent digital divide [23]. However, they also considered that current procedures for informing patients about potential uses of their data involve complicated and lengthy documents filled with jargon that can be incomprehensible during a typical visit and may be inappropriate in some circumstances, such as when a patient is receiving a cancer diagnosis. Participants also raised concerns about the uses of health data, expressing concerns about potential discriminatory practices, such as denial of life insurance.

### Comparison With Prior Work

Our study suggests the deliberative approach can be valuable for engaging patients and can inform policy making in a way that reflects patient perspectives on complex topics [20,24]. Unlike surveys, interviews, or focus groups, which often limit opportunities for sharing information with participants, our deliberations increased awareness about health data sharing among participants and, with extended periods of facilitated discussion, also provided insight into how participants reason and negotiate their individual needs and preferences with the benefits and risks to society [24,25]. As patients with experience of different types of cancer, participants in this study recognized that the policies discussed had the potential to impact future patients with cancer.

Precision oncology and related data-intensive technologies and methods such as AI are rapidly evolving and increasingly incorporated within the medical system. Decisions about ethical uses of the data needed for these technologies should be informed by public input and should reflect public health values, including consideration of the benefits and risks to society, as well as health equity [26,27]. This will become increasingly important as large data sets reflect the populations and communities proximate to the hospitals and health care systems utilizing their data in precision health, AI and machine learning, and learning health systems.

Public deliberation—engaging patients in discussions to understand their concerns and policy preferences—is a promising approach for soliciting this input. There are a variety of models for conducting public deliberations, with variation in how participants are recruited, how the policy issue is presented and framed, and what lens frames the deliberation (ie, whether it is led by policymakers or researchers) [28,29]. Public deliberation models also vary in the number of deliberators and the length of the deliberation. For example, one recent deliberation was conducted over two 2-day periods, while others engage fewer people in a single session [30]. While it is possible that participants’ perspectives may evolve over time and as they develop comfort with their fellow participants, our analysis found that there was positive engagement and candor in the groups in a short time period, comparable to other models [16,30]. Processes such as facilitator training were valuable, as they contributed to standardization in the conduct of small discussions across both sessions. The involvement and availability of experts was helpful for ensuring consistency in any additional information provided to participants (eg, in response to questions). While we conducted a debrief with facilitators following each session, conducting a formal evaluation may have generated further insights into the nature of discussions and any areas for improvement in future deliberations. Continuing to develop, evaluate, and systematically measure outcomes of these deliberative approaches is crucial for extending their utility in policy making.

### Limitations

Our descriptive study has some limitations. Public deliberation assumes people are comfortable voicing opinions in a group setting, which can exclude certain participants, particularly those who do not feel empowered or comfortable sharing [31]. Despite the use of facilitator training that, among other things, emphasized the importance of inclusivity, it is possible that there were differences in facilitator approaches, such as their tone and responses to deliberator comments and questions. Finally, as the deliberators were from one specific geographic region and were current or previous patients at the same institution, it is possible that they all had similar experiences they were drawing upon when grappling with complex policy options that were potentially different from patients from other health systems. Further, as the deliberators had current or previous cancer, it is possible that their concerns and preferences were different from those of the general public; that is, people without cancer or other chronic conditions. Nevertheless, our approach enabled us to gain rich insights into the different types of needs and concerns of patients with current or former cancer diagnoses and to elaborate on the utility of public deliberation as a method for gathering data about patient preferences and the rationale behind those preferences.

### Conclusion

The findings from our two deliberations—marked by the opportunity for education and informed dialogue—illustrate the value of deliberative approaches for soliciting patient concerns and preferences related to health data sharing and, by extension, other complex topics. The promise of health data sharing and learning health systems is contingent on patient trust and confidence that their health information is being used and shared in ways that meet their expectations. Using deliberative methods that provide information to patients and the opportunity to reason with complex information in accordance with public health values and the ideals of equity offers an important step for creating and nourishing patient trust and confidence.

## Appendix

Multimedia Appendix 1 Educational booklet.

Multimedia Appendix 2 Session agenda.

Multimedia Appendix 3 Post deliberation survey.

## Abbreviations

AI: artificial intelligence
EHR: electronic health record
