# Supplementary material for: Public Deliberation Process on Patient Perspectives on Health Information Sharing: Evaluative Descriptive Study
Source: JMIR Cancer. 2022 Sep 16;8(3):e37793. doi: 10.2196/37793 (PMC9526123; doi:10.2196/37793)
Supplement: Multimedia Appendix 1 [file cancer_v8i3e37793_app1.pdf]

Health Data Sharing:
A Public Deliberation

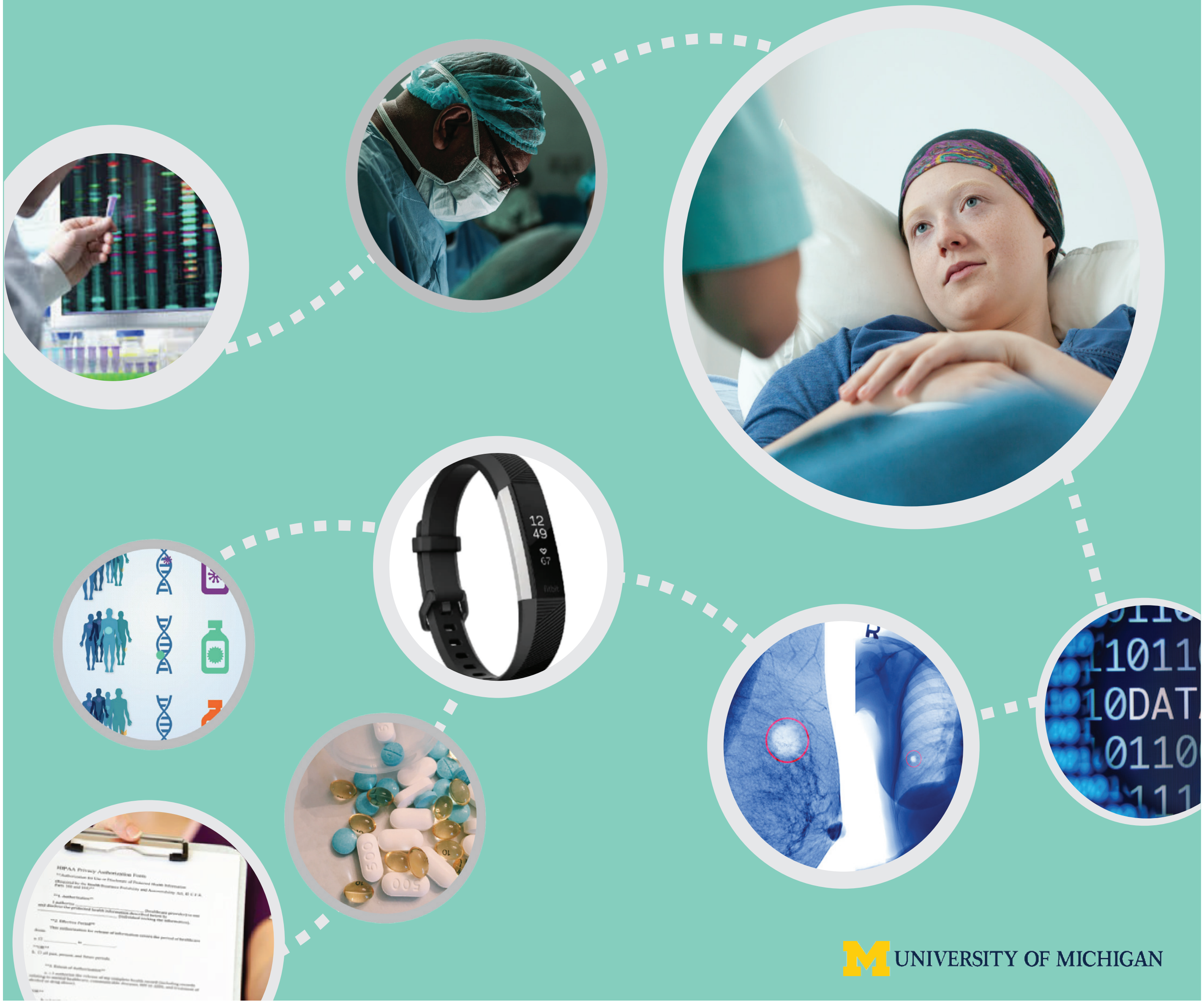

UNIVERSITY OF MICHIGAN

CONTENTS

YOUR DELIBERATION OVERVIEW 2

HEALTH DATA 3

WHO COLLECTS DATA? WHERE IS IT STORED? 4

SOME KEY TERMS 5

WHO USES HEALTH DATA? WHY? 6

LEARNING FROM HEALTH INFORMATION 7

DISEASE REGISTRIES 8

HOW HEALTH INFORMATION IS SHARED 9

WILL PEOPLE KNOW IT'S MY DATA? 10

HIPAA & ITS PRIVACY RULE 11

BENEFITS, RISKS AND ISSUES TO CONSIDER 12-15

THE FUTURE 16

GLOSSARY OF TERMS 17-18

WELCOME!

A public deliberation is a community discussion that brings people into the process of making decisions or solving problems that affect them.

This democratic process helps us work together to understand challenging issues and consider different perspectives.

For this project, participants will be considering how health data should be used, shared & regulated.

Thanks for being here!

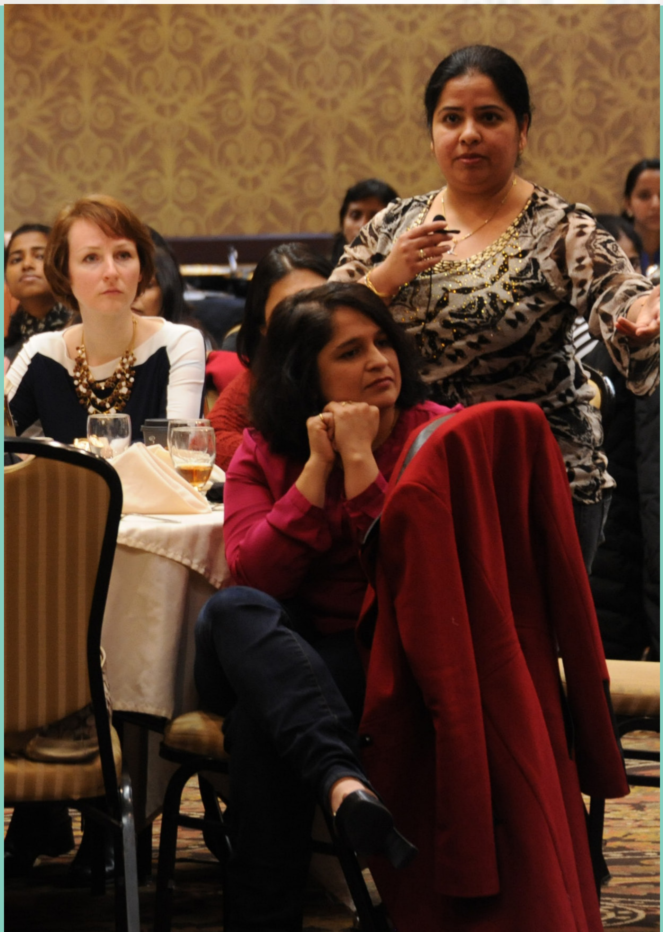

YOUR DELIBERATION OVERVIEW

During the deliberation, we'll hear about health data sharing from speakers who are experts in this area. You're not expected to be an expert on this topic, but to bring your opinions, values and ideas to the conversation. You will work together with other participants to make recommendations that can shape policies about how health data is used and shared.

KEY QUESTIONS

- WHAT POLICIES SHOULD GUIDE HOW HEALTH DATA IS USED & SHARED?
- WHAT SHOULD PEOPLE KNOW ABOUT HOW THEIR HEALTH DATA IS USED & SHARED?

GROUND RULES

- BE RESPECTFUL & LISTEN WELL
- KEEP AN OPEN MIND
- NO EYE-ROLLING
- AVOID CROSS-TALK
- TRY NOT TO INTERRUPT
- ASK QUESTIONS
- GIVE REASONS FOR YOUR OPINIONS
- YOU DON'T HAVE TO SHARE ANY INFORMATION ABOUT YOUR HEALTH HISTORY
- IF OTHERS SHARE PERSONAL INFORMATION, PLEASE DON'T SHARE IT OUTSIDE OF TODAY'S DELIBERATION

© 2019-2020 University of Michigan

HEALTH DATA

"Health data" comes in many forms.

- **BIOLOGICAL DATA** (E.G., GENETIC INFORMATION, HEIGHT/WEIGHT, BLOOD/TISSUE SAMPLE)
- **CLINICAL DATA** (E.G., BLOOD PRESSURE, TEST RESULTS)
- **TRACKING DATA** (E.G., GPS, DAILY ACTIVITY)
- **ADMINISTRATIVE DATA** (E.G., ADMISSIONS, BILLING RECORDS)
- **REGISTRY DATA** (E.G., BIRTH RECORDS, NATIONAL CANCER DATABASE)

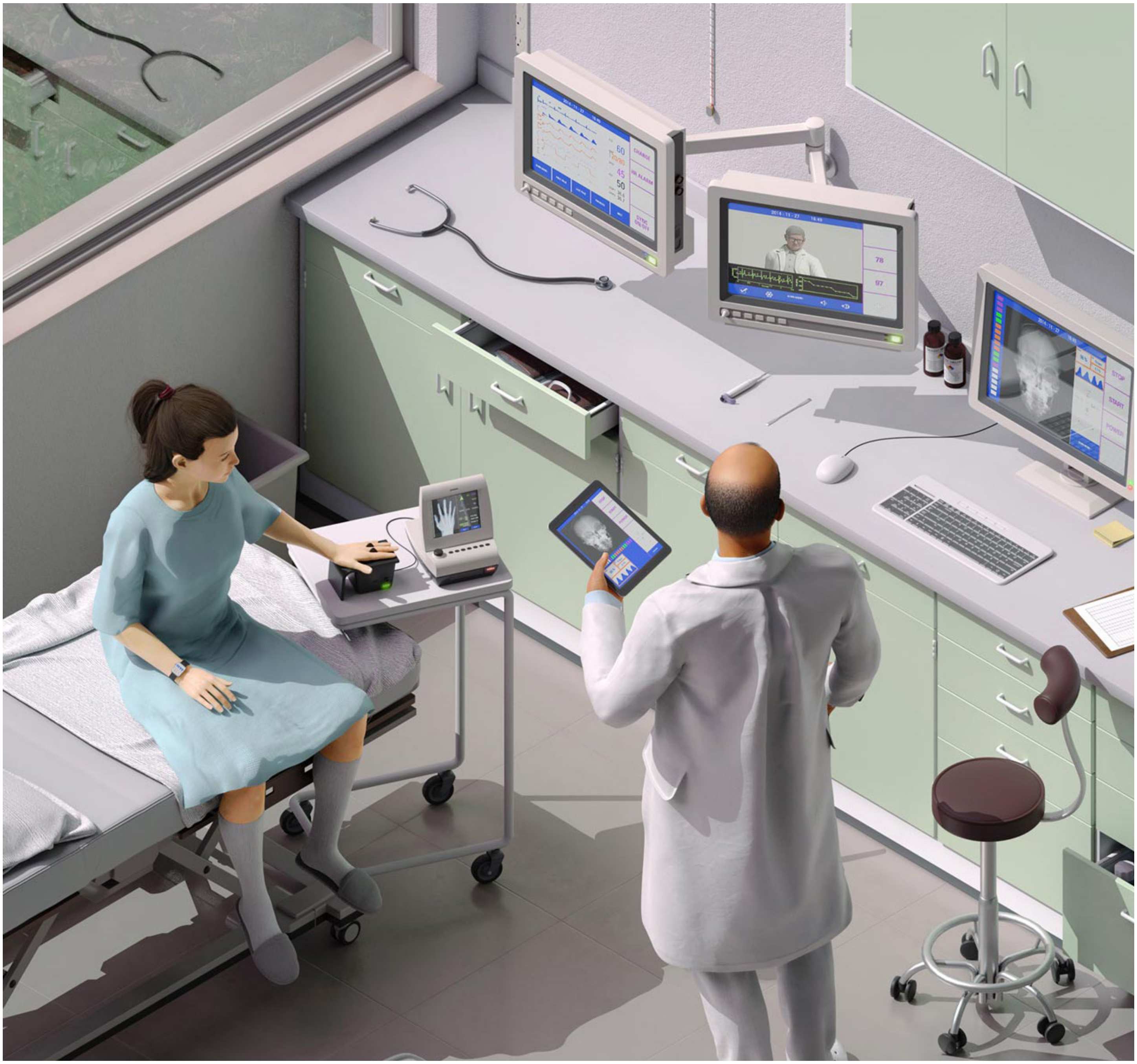

Illustration by Max Guther

WHO COLLECTS HEALTH DATA? WHERE IS IT STORED?

Where does your health data live? Health data is collected and stored by individuals and institutions-- locally (like in a clinic or hospital), state-wide, and in national databases.

A FEW EXAMPLES:

WHO?	WHAT?	WHERE?
Your physicians	Health history, prescriptions	Electronic Medical Record (EMR or EHR)
Hospitals	Health history, outcomes of cancer treatment	EMR, hospital cancer registry
Departments of Health	Immunization records, outbreaks, cancer data	CDC, health department, state cancer registry
Pharmaceutical & Medical Device Companies	Clinical trial data, tumor sample	Private company
Researchers	Surveys, studies	University
You	Diet journal, exercise stats	Fitbit, mobile health apps, online

Health data is fueling a large, growing industry. North America's market for "big data" in health care is expected to exceed $30 billion by 2025. To tap the value of vast stores of data, organizations are using new technologies to analyze them. Goals of this industry include supporting patient care, reducing healthcare costs, making money, and developing "personalized" or **"precision" medicine.**

© 2019-2020 University of Michigan

SOME KEY TERMS

Note: More key terms and definitions can be found in the glossary at the end of this booklet.

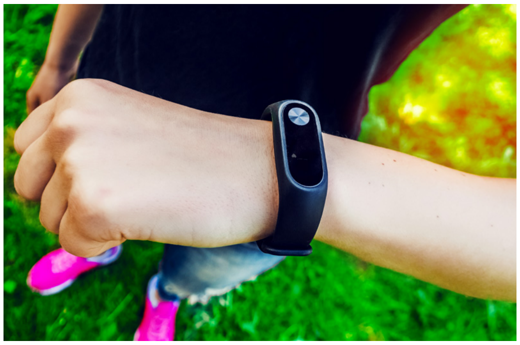

Individual-level health data is information about individual patients such as

- medical records
- blood test results
- exercise habits
- disease risk
- prescription medicines

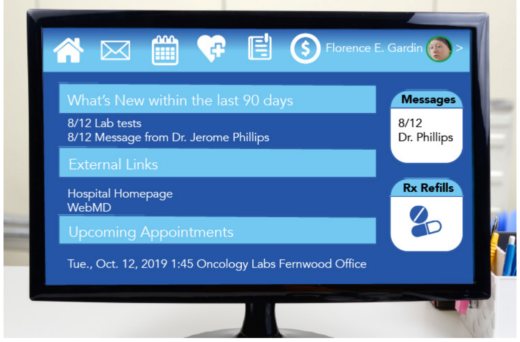

Today, individual health data is stored digitally on computers, and is easier than ever to use and share. Patients often use an online **patient portal** to access some of their health data, like

- recent doctor visits
- medications
- lab results
- other information in their **electronic medical record (EMR)**

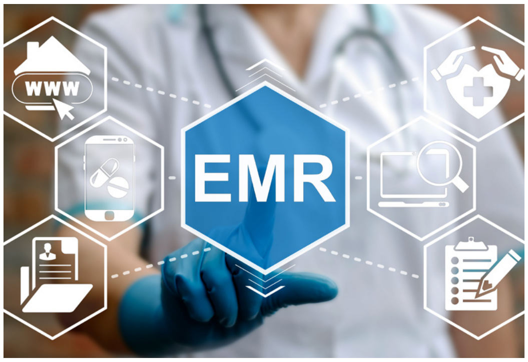

The answers to big questions in health care don't usually lie in a single person's medical record. Instead, they're found in *patterns* that can be detected when data from *many* people are put together.

When many different *kinds* of health data are combined, better discoveries and predictions can be made.

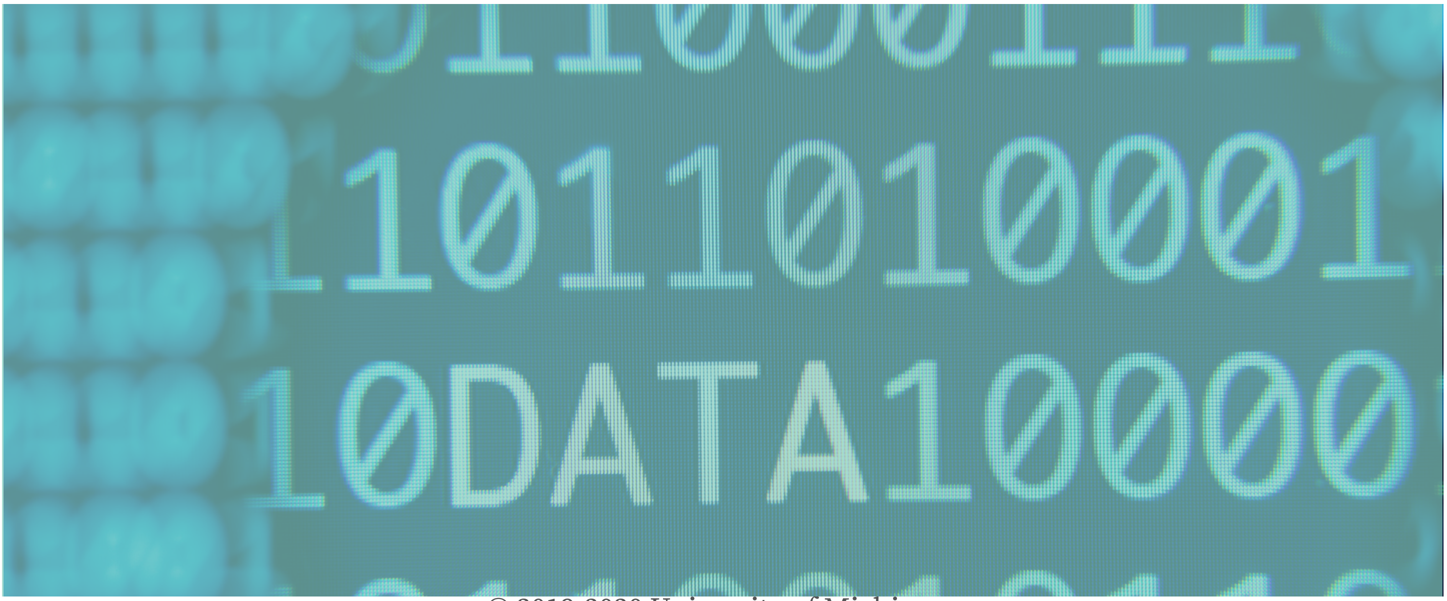

© 2019-2020 University of Michigan

WHO USES HEALTH DATA? WHY?

Health data is used for many purposes by different kinds of organizations.

A FEW EXAMPLES OF ORGANIZATIONS THAT USE HEALTH DATA:

Hospitals & Health Systems

Public Health Organizations

Insurance Companies

Pharmaceutical & Medical Device Companies

Genetic Testing Companies

Your data can be used to improve your care, like when:

- a doctor sees you're allergic to a medicine and doesn't prescribe it
- a doctor understands your family's history
- your clinic can send you an appointment reminder...

Your data can also be used to help *other patients*.

Health data is valuable for:

- public health
- research
- developing new drugs or treatments
- improving the quality of health care
- and more....

© 2019-2020 University of Michigan

LEARNING FROM HEALTH INFORMATION

Patient health information can be used in many ways that improve care for individuals and groups. This process can repeat in a loop as new patients share their data.

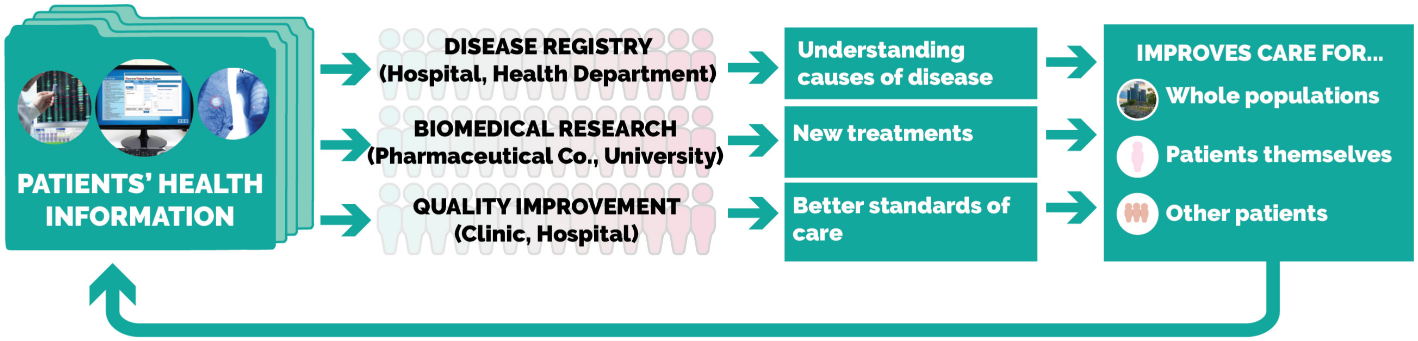

SPOTLIGHT:

The idea of "**precision medicine**" is to give the "right treatment to the right patient at the right time" by customizing their health care. A major focus of this approach is cancer prevention, diagnosis and treatment.

Genetic testing of healthy and unhealthy cells, for example, can sometimes reveal how a patient will respond to drugs, or help create new possibilities for treatment.

In our deliberations, we'll focus especially on how health care systems, public health organizations, and commercial companies use and share health data related to cancer.

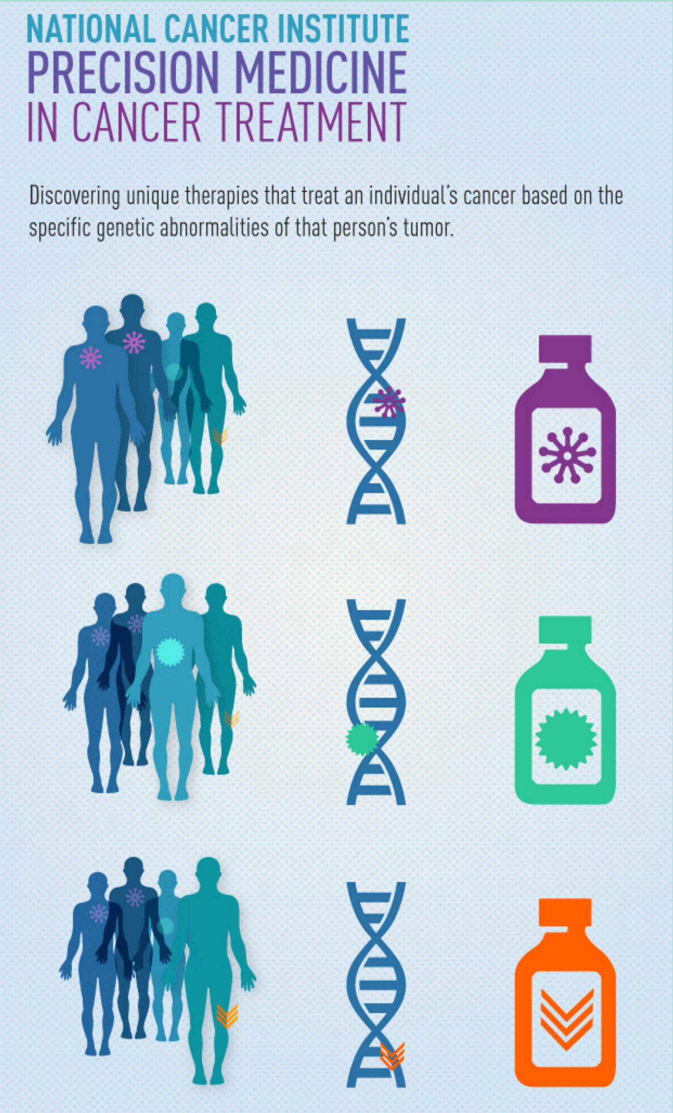

SOURCE: National Cancer Institute at the National Institutes of Health

© 2019-2020 University of Michigan

DISEASE REGISTRIES

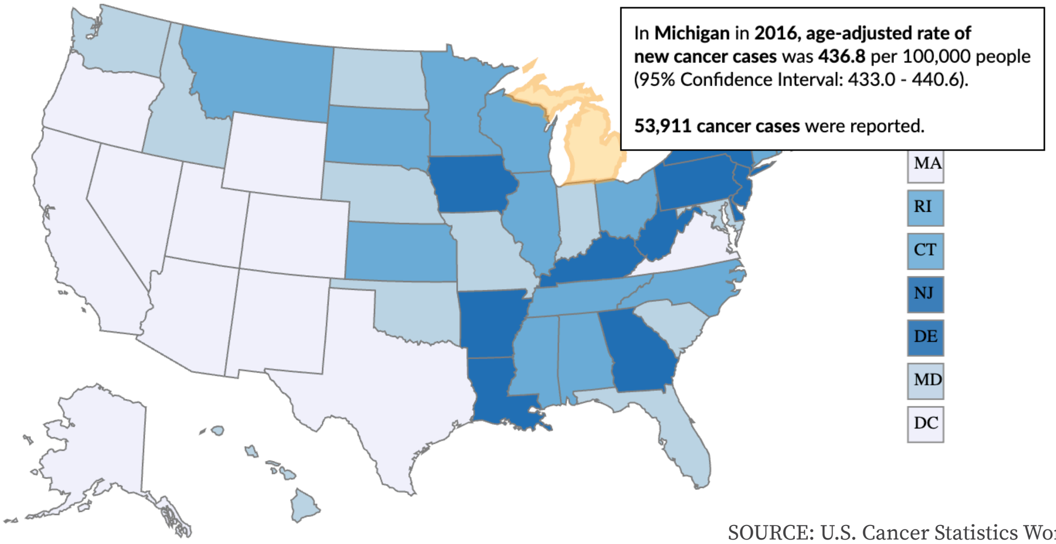

SOURCE: U.S. Cancer Statistics Working Group

Information about specific groups and certain health conditions are pooled together into disease databases or "registries."

Health information sharing or "reporting" of some diseases to public health agencies may be required to keep people safe. Examples of diseases that must be reported are:

- measles
- rabies
- plague
- sexually transmitted infections (STIs)

Decisions about how much health information should be tracked and shared often involve weighing factors like:

- the usefulness of the data
- the seriousness of the disease
- privacy
- personal rights
- treatment options
- costs

In some cases, patients have sought to limit how much information is reported for specific health issues. On the other hand, breast cancer survivors have been especially vocal about the importance of cancer registries.

SPOTLIGHT: A **cancer registry** collects detailed information about cancer patients and the treatments they receive and stores it in a searchable computer database. Hospital cancer registries keep data about the outcomes of treatment to improve patient care. **State and national registries** collect data to understand how cancer impacts large populations over time. They can answer questions like: Did lung cancer rates decrease this year? What groups are most likely to get skin cancer? What counties need more resources to support breast cancer screening?

HOW HEALTH INFORMATION IS SHARED

Most people in the U.S. have health information that is shared through their state's **Health Information Exchange (HIE).**

A Health Infomation Exchange allows health data to be shared electronically among "authorized users" such as:

- doctors, nurses
- insurance companies
- departments of health
- hospitals
- labs
- pharmacies
- Medicare
- possibly other HIEs or information networks.

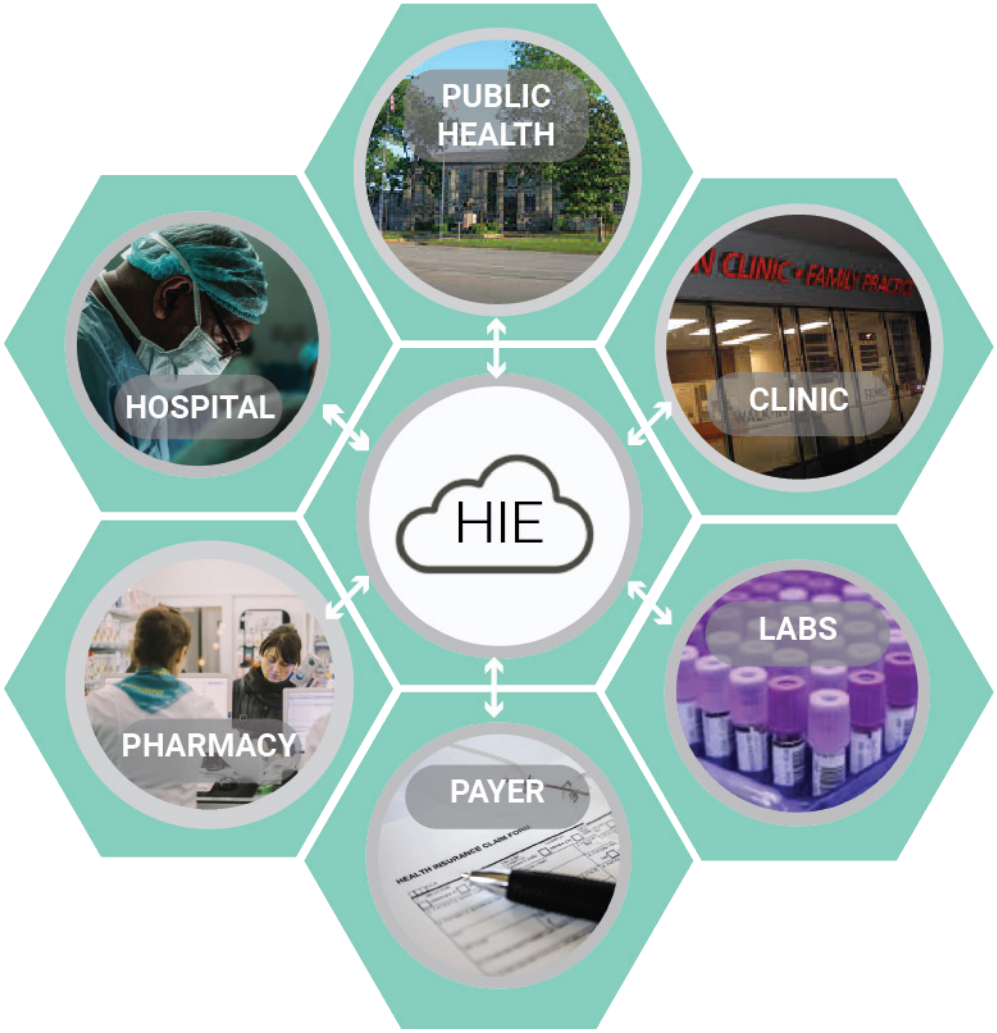

Health Information Exchanges help health professionals see more complete pictures of patients' records, such as:

- past visits
- medications
- CT scans
- other providers
- lab tests
- hospitalizations

This can help patients get the right care. The goal is to improve efficiency, reduce costs, improve safety, and monitor quality of health systems.

© 2019-2020 University of Michigan

WILL PEOPLE KNOW IT'S MY DATA?

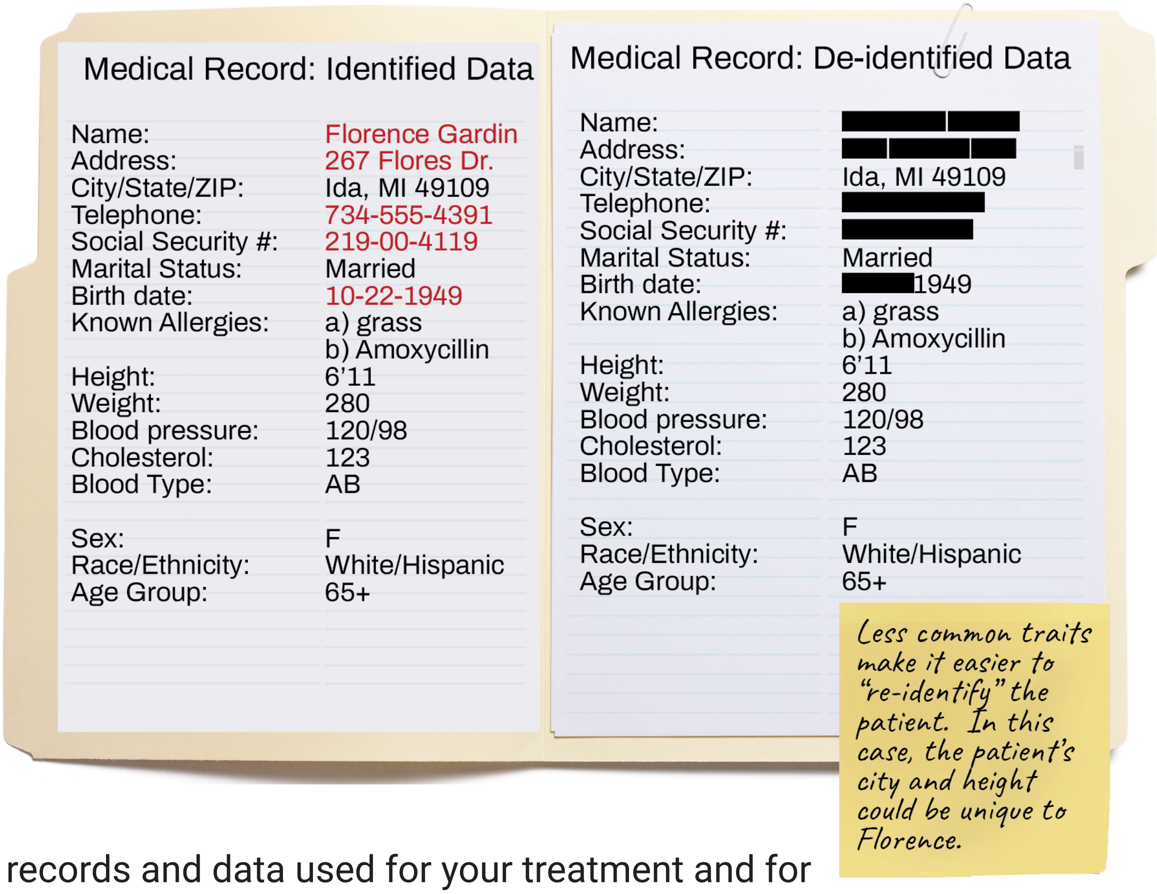

Health records and data used for your treatment and for **quality improvement** (e.g., your hospital's ability to "learn" from your experience) have your name and other "identifiers" attached. This is **identified data**.

In some situations, names and information that could identify you (like your phone number) are removed from health data before they're stored or shared. This is called **de-identified data**. Think of a legal document where "classified" information has been blacked out. Authorized individuals may have access to the "full report," or to a "key" that an organization keeps in case that data needs to be re-identified.

While unlikely, there's always a risk that data with names removed could still be **re-identified** if there's enough combined information for someone to narrow in on someone's unique profile. This is especially true when data includes DNA or genomic data.

Data is only **anonymous** when personal data has been irreversibly altered and there is no way to reidentify it.

© 2019-2020 University of Michigan

HIPAA & ITS PRIVACY RULE

Many federal, state, local and institutional policies & practices affect how health data is used and shared in different contexts. In the U.S., practices are largely guided by the **Health Insurance Portability and Accountability Act (HIPAA)**.

The HIPAA Privacy Rule was created to set limits about what information health care providers can and can't share without authorization from the patient. It also gives patients the right to get a copy of their health records, and to request corrections to them.

Under HIPAA rules, identified patient information can be shared for the purposes of treatment, payment, and operations (quality improvement). "**Quality improvement**" efforts are focused on evaluating and improving health care in a local setting such as a hospital, health system, or doctor's office.

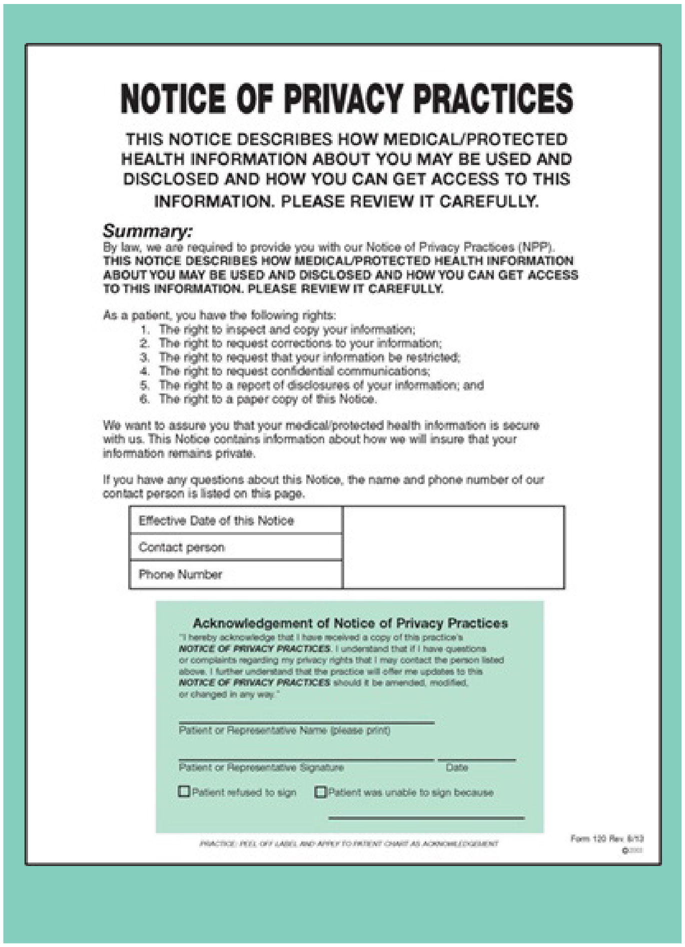

NOTICE OF PRIVACY PRACTICES

THIS NOTICE DESCRIBES HOW MEDICAL/PROTECTED HEALTH INFORMATION ABOUT YOU MAY BE USED AND DISCLOSED AND HOW YOU CAN GET ACCESS TO THIS INFORMATION. PLEASE REVIEW IT CAREFULLY.

Summary:
By law, we are required to provide you with our Notice of Privacy Practices (NPP). **THIS NOTICE DESCRIBES HOW MEDICAL/PROTECTED HEALTH INFORMATION ABOUT YOU MAY BE USED AND DISCLOSED AND HOW YOU CAN GET ACCESS TO THIS INFORMATION. PLEASE REVIEW IT CAREFULLY.**

As a patient, you have the following rights:

1. The right to inspect and copy your information;
2. The right to request corrections to your information;
3. The right to request that your information be restricted;
4. The right to request confidential communications;
5. The right to a report of disclosures of your information; and
6. The right to a paper copy of this Notice.

We want to assure you that your medical/protected health information is secure with us. This Notice contains information about how we will insure that your information remains private.

If you have any questions about this Notice, the name and phone number of our contact person is listed on this page.

Effective Date of this Notice	
Contact person	
Phone Number	

Acknowledgement of Notice of Privacy Practices
"I hereby acknowledge that I have received a copy of this practice's ***NOTICE OF PRIVACY PRACTICES***. I understand that if I have questions or complaints regarding my privacy rights that I may contact the person listed above. I further understand that the practice will offer me updates to this ***NOTICE OF PRIVACY PRACTICES*** should it be amended, modified, or changed in any way."

Patient or Representative Name (please print)

Patient or Representative Signature   Date

☐ Patient refused to sign   ☐ Patient was unable to sign because

PRACTICE: PEEL OFF LABEL AND APPLY TO PATIENT CHART AS ACKNOWLEDGEMENT

Form 120 Rev. 8/13

If you've received health care in the U.S. in the past 16 years, you've probably signed a HIPAA form.

Your signature is just an acknowledgement that you received information about HIPAA, including how your provider can use and share your health information. It doesn't affect how your health information is shared.

HIPAA doesn't regulate most:

- health information that a patient shares on the internet, or
- health data that is collected by a **private company**.

If you have an app tracking your diet or exercise:

- that information might not be covered by HIPAA, and
- companies can share or sell that information

© 2019-2020 University of Michigan

BENEFITS, RISKS & ISSUES TO CONSIDER

Health data sharing has enormous potential to answer major health questions and to improve people's health and health care. But there are tradeoffs to consider. Health data sharing raises questions about issues like privacy, security, transparency, equity, and fairness...

ETHICS

Who should be allowed to see personal health information? Will it only be used for good? Is there an obligation for people to share their health information, if doing so could save lives? Are there enough laws and protections in place to protect sensitive information? Health information sharing raises many questions about what is right. Thinking about the future of health information sharing involves thinking through consequences that could be good, bad, or unintended.

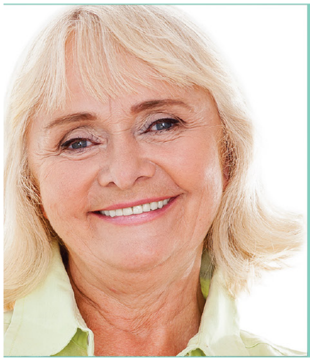

"I'd like more control over how my health information is shared. Having choices is a sign of respect."

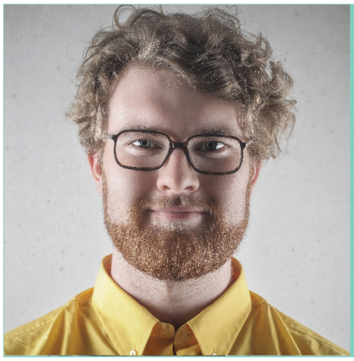

"When I learned that my tumor sample might be used to help patients like me, it made me feel good."

TRUST | TRANSPARENCY

Data sharing and use that goes beyond patients' intentions or expectations could generate mistrust. Institutions may fall short of providing basic information to patients about quality improvement programs, sharing information with private companies, or even findings that could be relevant to their own clinical care or could affect their health. Organizations that fail to be transparent about health information sharing risk backlash from individuals if they feel their trust or privacy has been violated.

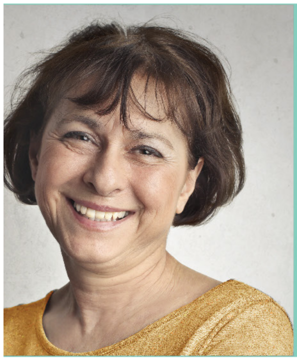

"People who are working to solve health problems will do the right thing with my data. I don't really have time to worry about it."

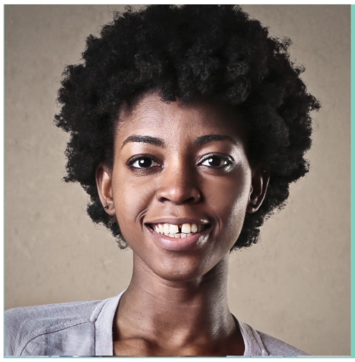

"It's a matter of trust. People have to be told about how their information is used. My community tends to be the last to know and the last to benefit."

© 2019-2020 University of Michigan

BENEFITS, RISKS & ISSUES TO CONSIDER

PRIVACY

People have different levels of comfort about sharing their health information, especially when it's identified. Even when health data are de-identified, with enough information, resources and effort, there is a possibility that they could be **re-identified**, or traced back to specific individuals.

Decisions about health data sharing often involve weighing privacy risks against potential benefits. There may be a trade-off between the ability to conduct research and treat patients, and protecting the privacy of people in the data.

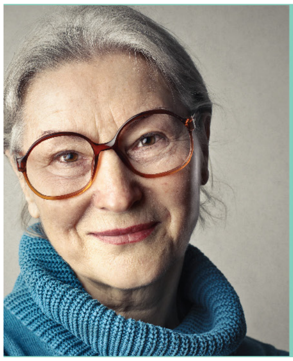

"My health information is mine. I should decide who sees it, and how it is shared."

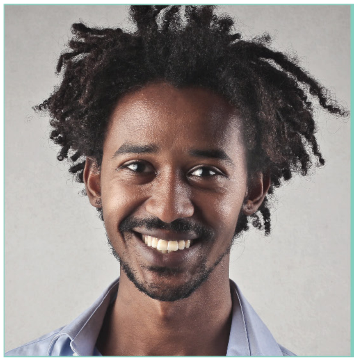

"My information is already out there on Facebook, Google, and the Internet. Health information is the same."

EQUITY | SOCIAL JUSTICE

The handling of health information can raise issues related to social justice, including discrimination and stigma. For example, if only people who can afford health insurance can get a certain kind of genetic test for their disease, then the information collected about the disesase will routinely leave out groups of people who can't afford health insurance. Even when we discover great new things in research, data biases might mean that only certain types of communities might benefit from that research.

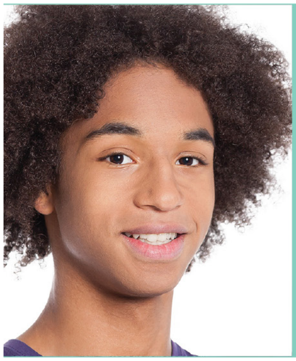

"I don't think people and companies that are using my health information really care much about me. I doubt any of this will help me or my family."

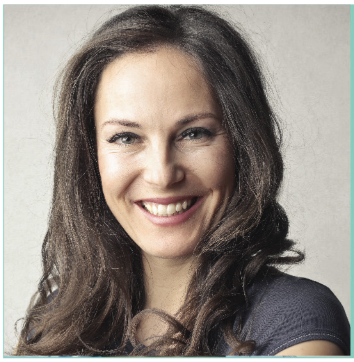

"I'm afraid my health information could be used against me."

© 2019-2020 University of Michigan

BENEFITS, RISKS & ISSUES TO CONSIDER

SECURITY

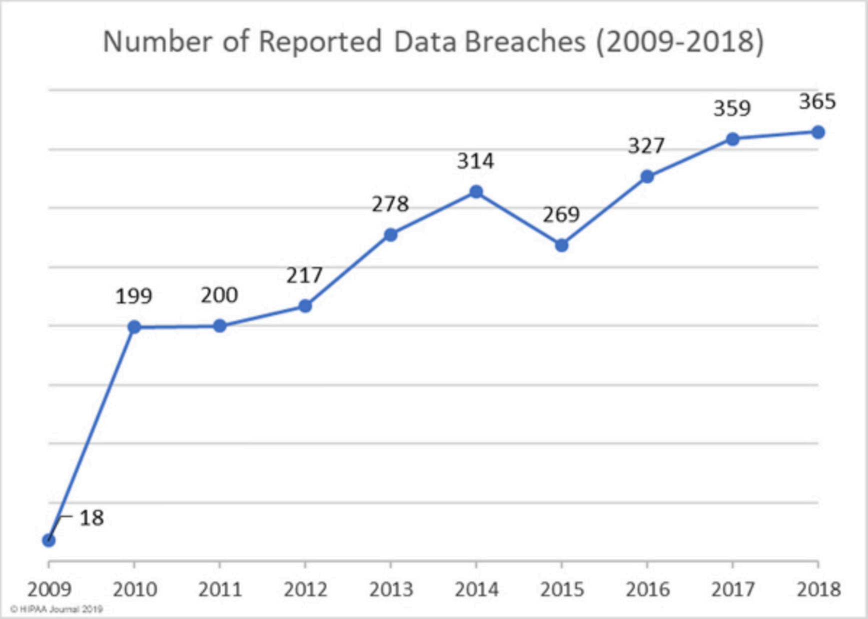

One major risk to privacy, called a "data breach," happens when sensitive data is lost, stolen, hacked or improperly shared.

In the last decade there's been an upward trend in the frequency of healthcare data breaches.

Between 2009 and 2018 there have been more than 2,500 healthcare data breaches that have resulted in the theft or exposure of ~190 million healthcare records. That equates to more than 59% of the U.S. population.

Recent breaches have most commonly been caused by unauthorized access, sharing or hacking of health data stored by healthcare providers.

While many breaches involve little risk to patients, such as the accidental disclosure of a name, the consequences can sometimes be severe. Many victims of a major breach by Anthem Inc. in 2015 had tax returns filed in their names, resulting in financial losses. Others have been victims of medical identity theft or lost health care coverage.

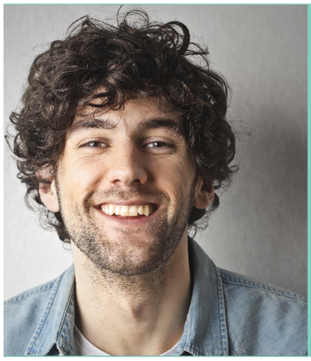

"Of course I expect my hospital will keep my information safe. If we can't trust doctors with our data, who can we trust?"

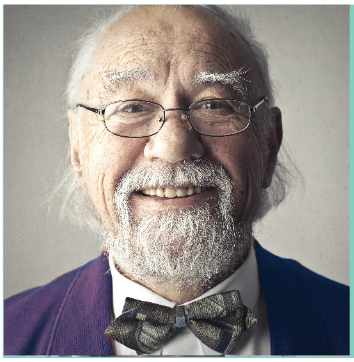

"Meh, it's a small risk someone would steal my informa-tion and an even smaller risk anyone would care what it said."

© 2019-2020 University of Michigan

COMMERCIALIZATION

Health care innovation, and in particular, storing human biospecimens (like blood) for future use is expensive. One way that hospitals might cover these costs is by "**commercializing**" the health data: that is, selling it to private, for-profit companies (e.g., drug companies.) This could lead to important strides for developing treatments. But we also know that people might be uncomfortable when their local hospital sells their data or biospecimens to private companies. How can hospitals balance their need to support systems to keep health data and biospecimens safe with respecting people's privacy and preferences? What happens when decisions about health information sharing are financially motivated?

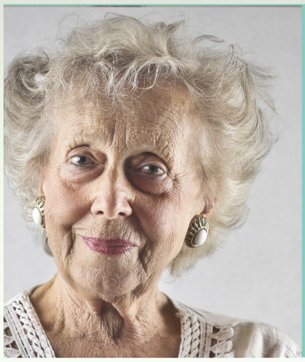

"It makes me uncomfortable to think that someone out there is making money off of my personal health information."

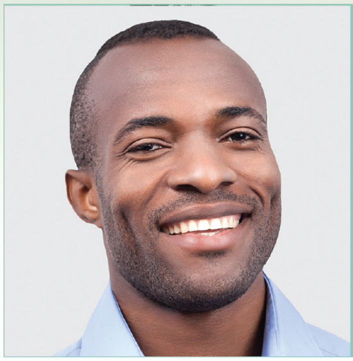

"Pharmaceutical companies need to make money to discover new treatments. I'm happy for my health info to be a part of that."

THE FUTURE

Technologies and laws that affect health care and privacy are constantly changing. New data protections are currently being discussed to address such issues as:

- notifying people about data sharing policies in plain language
- giving consumers more access and control over their data
- increasing penalties for health data breaches

Future health laws may do more to protect patient privacy or involve patients in health sharing practices. It's also likely that future laws will make it easier for organizations to share health care information or to help make health data more reliable and useful.

Public input will help guide the path ahead.

GLOSSARY OF TERMS

Administrative data: data collected in the course of providing and/or paying for services (e.g. hospital admissions, physician payment information)

Biospecimens: Biological samples from a patient (e.g., blood, tissue, urine)

Clinical data: detailed information about specific aspects of persons, conditions and/or care (e.g. blood pressure, weight, lab results)

Commercial companies: a company that is organized to make a profit

Commercialization: the sharing of health information for the purpose of making money

Data breach: an unauthorized release of data either through hacking or by accident

De-identified data: data where the personal identifiers of the individuals have been removed

Electronic health record (EHR) or electronic medical record (EMR): health histories collected from patient visits to clinics and hospitals that can be shared electronically

Individual-level data: data that are collected from individuals and that can be associated to an individual

Health Data: any type of information that contains information about the health conditions of a person or group of people. "Health data" might include biospecimens (e.g., blood/tissue samples)

Health provider: a person or company that takes care of patients

Healthcare system: an organized group of people, institutions, and resources that deliver health care services to meet the health needs of populations

Health Information Exchange: the electronic transfer of a patient's medical information between doctors, nurses, pharmacists, other providers, and patients

HIPAA Privacy Rule: a rule that sets limits about what information can and can't be shared without authorization from the patient. It also gives patients the right to get a copy of their health records, and to request corrections

© 2019-2020 University of Michigan

GLOSSARY OF TERMS

Informed consent: A contract agreement that's meant to ensure that research subjects and patients have made a voluntary decision to participate in research after its purpose, risks and benefits have been fully explained

Notification: The alerting of patients to uses of their health data so that they're aware of it

Precision Medicine: an approach to treatment that takes into account individual or "personal" characteristics, like genes, environment or lifestyle

Private company: any privately owned business, corporation or enterprise

Public agencies: federal, state, and local organizations, including public health
organizations and agencies

Public deliberation: a community discussion that can inform policy decisions on issues that affect members of the public

Public health organizations: Groups tasked with preventing disease, prolonging life, and promoting health locally (e.g. in a county), nationally (e.g. in a country) or globally (around the world)

Quality improvement: the use of health data to measure "what works" to improve how care is delivered to patients within an institution

Reidentified data: Health information that has been traced back to an individual after it's been de-identified.

Registry data: Information about specified populations or health conditions (e.g., birth and death records, national cancer data base)

Research: the systematic investigation into and study of materials and sources in order to establish facts and reach new, generalizable conclusions

Survey data: information collected directly from and about individuals or groups

Tracking data: data that exists about individuals in the context of everyday life, (e.g. Fitbit, web activity, GPS tracking

© 2019-2020 University of Michigan

THIS BOOKLET WAS PREPARED BY THE LIFECYCLE OF DATA RESEARCH TEAM AT THE UNIVERSITY OF MICHIGAN.

Listed Alphabetically:
Karen Calhoun
Melissa Creary
Raymond De Vries
S.L.R. Kardia
Paige Nong
Jodyn Platt
Tevah Platt
Mina Raj
Kerry Ryan
Kayte Spector-Bagdady
Amanda Stanhaus
Daniel Thiel
Gracie Trinidad

This public deliberation is supported by the NIH research grant 5R01CA214829-02.

THE CONTRIBUTORS GRATEFULLY ACKNOWLEDGE THE BRITISH COLUMBIA DATA DELIBERATION RESEARCH TEAM FOR PROVIDING AN EXEMPLARY MODEL OF DELIBERATION ON DATA RESEARCH USE.

UNIVERSITY OF MICHIGAN
