# Supplementary material for: Public Deliberation Process on Patient Perspectives on Health Information Sharing: Evaluative Descriptive Study
Source: JMIR Cancer. 2022 Sep 16;8(3):e37793. doi: 10.2196/37793 (PMC9526123; doi:10.2196/37793)
Supplement: Multimedia Appendix 2 [file cancer_v8i3e37793_app2.docx]

# Session Agenda

| **Time** | **Duration** | **Activity & Topic** |
| --- | --- | --- |
| 8:15 am |  | **Registration Table opens** |
| 8:30-9:05am | 35 min | **Registration and First Survey**   - Breakfast - Pre-deliberation survey |
| 9:05-9:15am | 10 min | **Welcome and Introductions**   - Introduction of study team - Overview of deliberation day - Location/facilities information |
| 9:15-9:30am | 15 min | **“Ice Breaker” Small Group**   - Brief introductions |
| 9:30-10:15am | 45 min | **Plenary Session 1: Scenario A**   - What is precision oncology? - What kind of information is collected and shared? - What policies are in place to protect this information use? - What are the ethical concerns? - Intro to Scenario A & small group task |
| 10:15-10:30am | 15 min | **Q & A session**   - With Plenary Session 1 presenters |
| 10:30-10:45am | 15 min | **Break** |
| 10:45am-11:45am | 60 min | **“Scenario A” Small Group Session**   - Health Information Exchanges discussion - Voting on policy options for Scenario A - Discussion of “Tradeoffs” -- risks and benefits to individuals, families, communities and society |
| 11:45am-12:30pm | 45 min | **Lunch** |
| 12:30-1:00pm | 30 min | **Plenary Session 2: Scenario B**   - What information is shared outside the institution? - How is health information commercialized? - What are the policy issues and ethical concerns? - Intro to Scenario B & small group task |
| 1:00-1:15pm | 15 min | **Q & A session**   - With Plenary Session 2 presenters |
| 1:15-2:15pm | 60 min | **“Scenario B” Small Group Session**   - Commercial company involvement discussion - Voting on policy options for Scenario B - Discussion of “Tradeoffs” -- risks and benefits to individuals, families, communities and society |
| 2:15-2:30pm | 15 min | **Break** |
| 2:30-3:30pm | 60 min | **Large Group Review**   - Small groups share rankings (for Scenario A & B) - Policy discussion |
| 3:30-4:00pm | 30 min | **Second Survey & Check-out**   - Post-deliberation survey - Participants return 2nd survey to registration desk to check out |
