# Supplementary material for: Public Deliberation Process on Patient Perspectives on Health Information Sharing: Evaluative Descriptive Study
Source: JMIR Cancer. 2022 Sep 16;8(3):e37793. doi: 10.2196/37793 (PMC9526123; doi:10.2196/37793)
Supplement: Multimedia Appendix 3 [file cancer_v8i3e37793_app3.pdf]

Survey Instructions

This is a survey about your healthcare experience and your opinions about how health information is used and shared. We are interested in your thoughts and ideas.

We all use the healthcare system or know people who do. This system includes the healthcare provider, like a doctor or nurse, who you visit when you're sick or for routine visits. It also includes hospitals and people who work on quality improvement, and administrators who make decisions about how clinics and hospitals are run.

When we ask about "the healthcare system" we are asking generally about the healthcare system in this country. When we ask about "your healthcare system" we are asking about the healthcare professionals and institutions that you personally interact with.

Please answer each question to the best of your ability, but you may choose not to answer any questions you don't want to answer.

Scenario A

1. After reviewing the four “policy options” for Scenario A, how would you rank them in order of how strongly you support them (#1 being the one you support the most, and #4 being the least).
   A. Plain-language policy notification (Rank 1-4): ______
   B. Patient notification of health data sharing (text) (Rank 1-4): ______
   C. Patient access to health information sharing (portal) (Rank 1-4): ______
   D. No change to the current policy (Rank 1-4): ______

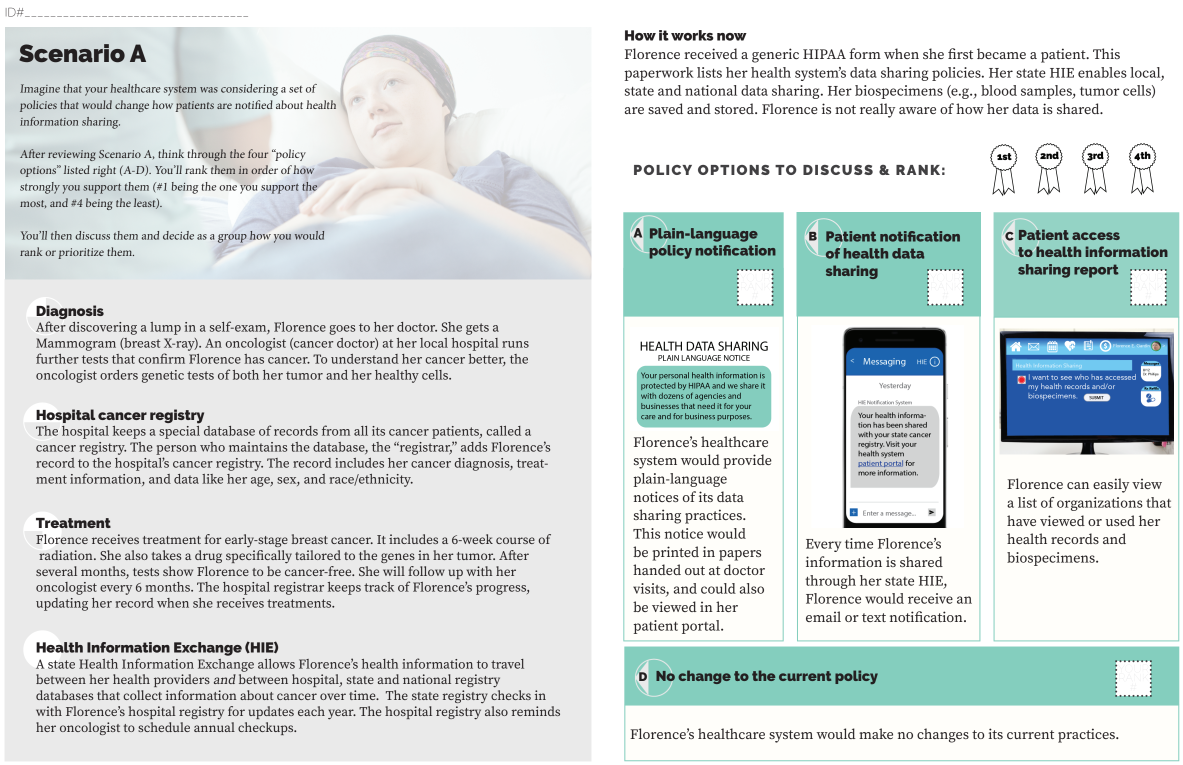
ID#______________________________

Scenario A

Imagine that your healthcare system was considering a set of policies that would change how patients are notified about health information sharing.

After reviewing Scenario A, think through the four “policy options” listed right (A-D). You’ll rank them in order of how strongly you support them (#1 being the one you support the most, and #4 being the least).

You’ll then discuss them and decide as a group how you would rank or prioritize them.

Diagnosis
After discovering a lump in a self-exam, Florence goes to her doctor. She gets a Mammogram (breast X-ray). An oncologist (cancer doctor) at her local hospital runs further tests that confirm Florence has cancer. To understand her cancer better, the oncologist orders genetic tests of both her tumor and her healthy cells.

Hospital cancer registry
The hospital keeps a special database of records from all its cancer patients, called a cancer registry. The person who maintains the database, the “registrar,” adds Florence’s record to the hospital’s cancer registry. The record includes her cancer diagnosis, treatment information, and data like her age, sex, and race/ethnicity.

Treatment
Florence receives treatment for early-stage breast cancer. It includes a 6-week course of radiation. She also takes a drug specifically tailored to the genes in her tumor. After several months, tests show Florence to be cancer-free. She will follow up with her oncologist every 6 months. The hospital registrar keeps track of Florence’s progress, updating her record when she receives treatments.

Health Information Exchange (HIE)
A state Health Information Exchange allows Florence’s health information to travel between her health providers *and* between hospital, state and national registry databases that collect information about cancer over time. The state registry checks in with Florence’s hospital registry for updates each year. The hospital registry also reminds her oncologist to schedule annual checkups.

How it works now
Florence received a generic HIPAA form when she first became a patient. This paperwork lists her health system’s data sharing policies. Her state HIE enables local, state and national data sharing. Her biospecimens (e.g., blood samples, tumor cells) are saved and stored. Florence is not really aware of how her data is shared.

POLICY OPTIONS TO DISCUSS & RANK: 1st 2nd 3rd 4th

A Plain-language policy notification

HEALTH DATA SHARING
PLAIN LANGUAGE NOTICE
Your personal health information is protected by HIPAA and we share it with dozens of agencies and businesses that need it for your care and for business purposes.

Florence’s healthcare system would provide plain-language notices of its data sharing practices. This notice would be printed in papers handed out at doctor visits, and could also be viewed in her patient portal.

B Patient notification of health data sharing

Messaging HIE
Yesterday
HIE Notification System
Your health information has been shared with your state cancer registry. Visit your health system patient portal for more information.
Enter a message...

Every time Florence’s information is shared through her state HIE, Florence would receive an email or text notification.

C Patient access to health information sharing report

Health Information Sharing
I want to see who has accessed my health records and/or biospecimens. SUBMIT

Florence can easily view a list of organizations that have viewed or used her health records and biospecimens.

D No change to the current policy

Florence’s healthcare system would make no changes to its current practices.

	Not at all	Somewhat	Fairly	Very much
2. How strongly do you feel about your ranking?	1	2	3	4
3. How much did your group discussion influence your ranking?	1	2	3	4
4. Do you have any comments about your ranking for Scenario A?				

Scenario B

5. After reviewing the five “policy options” for Scenario B, how would you rank them in order of how strongly you support them (#1 being the one you support the most, and #5 being the least).
   A. Commercial disclosure (portal) (Rank 1-5): ______
   B. Notification of health data sharing (text) (Rank 1-5): ______
   C. Opt out (Rank 1-5): ______
   D. Patient compensation ($) (Rank 1-5): ______
   E. No change to the current policy (Rank 1-5): ______

ID#________________________________

Scenario B

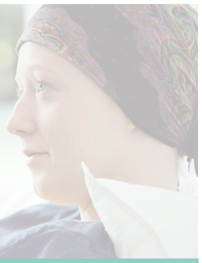

Imagine your healthcare system was considering a set of policies that would change how it shares health information with commercial companies and how patients are informed or involved when it does.

After reviewing Scenario B, think through the five “policy options” listed here (A-E). You’ll be asked to rank them in order of how strongly you support them (#1 being the one you support most, and #5 being the least).

You’ll then discuss them and decide as a group how you would rank or prioritize them.

Genetic Testing
Florence’s oncologist (cancer doctor) thinks that she might benefit from tailored treatments that block the growth and spread of cancer by interfering with specific molecules. To find out if this approach would work, samples of her tumor as well as her healthy cells are collected for genetic (DNA) testing.

Commercial lab
Florence’s hospital doesn’t have the expensive equipment or patent rights needed to do the genetic testing. So, they send Florence’s tumor and healthy cell samples to a commercial company that can do it-- Genom11. Genom11 sends the results back to Florence’s doctor. Florence does not know that her sample was sent outside of her local hospital.

Future uses of data & samples
To reduce the cost of genetic testing, the hospital has an agreement that Genom11 can keep tumor samples and her healthy DNA after testing. Genom11 can use samples and patient data for their own studies and to work on developing new drugs and treatments.

Profits
Genom11 owns thousands of samples. Genom11 can sell them to other companies and/or use them to develop new drugs and treatments. Genom11 makes money from selling samples, patient data, and products.

How it works now
Florence’s hospital has sharing agreements that allow commercial labs or companies to store data or samples and use them for ongoing purposes. The data sharing and privacy documents that patients receive do not include information about commercial companies that do genetic testing. Patients and their doctors will receive results of the the genetic tests that may help in their cancer treatment. However, patients do not receive any money for sharing their data or samples with commercial companies.

POLICY OPTIONS TO DISCUSS & RANK: 1st 2nd 3rd 4th 5th

A Commercial disclosure YOUR RANK #

Florence can use her patient portal to see a list of companies that have accessed her data and what data (including samples) they are permitted to keep.

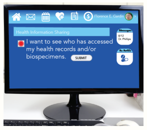

B Notification of data sharing YOUR RANK #

Florence’s hospital must notify her when her data is sold or shared with commercial companies.

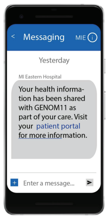

C Opt out YOUR RANK #

Florence can opt-out of the sharing or sale of her data with commercial companies. Opting out will not affect her care.

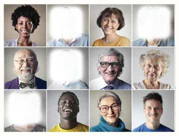

D Patient compensation YOUR RANK #

Florence receives payment (~$10) when her data is accessed or used by a commercial company.

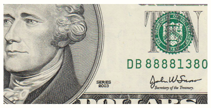

E No change to the current policy YOUR RANK #

When it comes to hospitals and businesses using her data and samples, Florence does not need to be more involved than she already is.

	Not at all	Somewhat	Fairly	Very much
6. How strongly do you feel about your ranking?	1	2	3	4
7. How much did your group discussion influence your ranking?	1	2	3	4
8. Do you have any comments about your ranking for Scenario B?				

The next questions are about the use of your health information by quality analysts.

Quality analysts are people who work for hospitals or clinics. They use patient health information at their hospital or clinic to check on, and improve, how their organization is working. They often study the cost of healthcare, their organization's efficiency on things like waiting room times, and the health of patients at their hospital or clinic.

9. For you, how true are the following statements? **Circle one** answer on each row.

	Not true	Somewhat true	Fairly true	Very true
A. I am comfortable with quality analysts using my *de-identified* health information.	1	2	3	4
B. I would like to be notified if quality analysts will use my *de-identified* health information.	1	2	3	4
C. I am comfortable with quality analysts using my *identified* health information.	1	2	3	4
D. I would like to be notified if quality analysts will use my *identified* health information.	1	2	3	4

The next questions are about the use of your health information by commercial companies.

Commercial companies are third-party companies that are not part of a hospital. For example, a third-party commercial company may conduct genetic tests and analyze information for a hospital or healthcare provider for a fee when a hospital is not able to conduct the test on their own. Commercial companies may keep the information for their own use.

10. For you, how true are the following statements? **Circle one** answer on each row.

	Not true	Somewhat true	Fairly true	Very true
A. I am comfortable with commercial companies using my *de-identified* health information.	1	2	3	4
B. I would like to be notified if commercial companies will use my *de-identified* health information.	1	2	3	4
C. I am comfortable with commercial companies using my *identified* health information.	1	2	3	4
D. I would like to be notified if commercial companies will use my *identified* health information.	1	2	3	4

11. How confident are you that *de-identifying* health information protects your privacy?

Not confident	Somewhat confident	Fairly confident	Very confident
1	2	3	4

The next questions are about the use of your DNA by commercial companies.

Suppose you are a cancer patient at a leading cancer center and your healthcare provider wants to use your DNA to see if you might be a good candidate for a particular cancer treatment. Your cancer center shares DNA and health information with third-party commercial companies when it is unable to perform the analysis themselves.

12. How comfortable are you with a third-party commercial company…?

	Not comfortable	Somewhat comfortable	Fairly comfortable	Very comfortable
A. …using your DNA and health information to improve the diagnosis and treatment of cancer in *other* patients.	1	2	3	4
B. …developing predictions about how you will respond to a particular cancer treatment.	1	2	3	4
C. …storing your DNA and health information.	1	2	3	4
D. …sharing predictions about how you will respond to cancer treatment with insurance companies.	1	2	3	4
E. …selling de-identified health information to pharmaceutical companies.	1	2	3	4

The next questions are about organizations that have your health information.

Organizations include groups such as healthcare providers' offices, hospitals, insurance companies, and university researchers. (If you are unsure, please make your best guess.)

13. The organizations that have my health information and share it...

	Not true	Somewhat true	Fairly true	Very true
A. … have a particular interest in collecting my biospecimens compared to other people's	1	2	3	4
B. …experiment on patients without telling them	1	2	3	4
C. … treat everyone the same, regardless of their race or ethnicity	1	2	3	4
D. … treat everyone the same, regardless of their income	1	2	3	4

The next questions are about healthcare providers and healthcare systems.

14. For you, how true is the following statement about health care providers?

All things considered, health care providers in this country can be trusted.

Not true	Somewhat true	Fairly true	Very true
1	2	3	4

15. For you, how true are the following statements? **Circle one** answer on each row.

	Not true	Somewhat true	Fairly true	Very true
A. I am satisfied with the level of access I have to information in my electronic health record.	1	2	3	4
B. My healthcare system respects my privacy.	1	2	3	4
C. My healthcare system discloses its conflicts of interest.	1	2	3	4
D. My healthcare system will use my health information how they see fit, regardless of my preferences.	1	2	3	4
E. It is important that I know who has health information about me.	1	2	3	4

The next questions are about how profits should be used by your healthcare system.

Suppose your healthcare system charges a fee to third-party commercial companies that want to keep biospecimens (like blood, tissue, or urine) left over after testing. This makes money for the hospital.

For you, how true are the following statements?

16. If my healthcare system makes money from my biospecimens…

	Not true	Somewhat true	Fairly true	Very true
A. …they should use the money to provide health care for other people who can't afford it.	1	2	3	4
B. …they should use the money to provide health care for other people who have the same health problems as me.	1	2	3	4
C. …they should use the money to improve quality of care in my healthcare system.	1	2	3	4
D. …they should use the money to support future research on my health problems.	1	2	3	4
E. …they should use the money to support future research on any kind of health problem.	1	2	3	4
F. …they should use it however they want.	1	2	3	4

17. **Circle** one answer for each statement below:

A. Your physician determines all uses of information in your medical record.

True False Not Sure

B. Only healthcare providers can access medical records.

True False Not Sure

C. State and local health departments collect information from physicians and clinics to monitor the health of communities.

True False Not Sure

D. Current health privacy laws prevent private companies from buying or accessing your health information.

True False Not Sure

E. My healthcare provider uses an electronic health record.

True False Not Sure

F. I have used a patient portal to access my health information online.

True False Not Sure

Circle one answer:

18. How much do you know about how health information is shared in the health system today?

Very little				A lot
1	2	3	4	5

19. How does your knowledge of health information sharing compare to what other people know?

Much lower than others		Equal to others		Much higher than others
1	2	3	4	5

The next questions are about your views of health information sharing.

20. **Circle** one answer:

    A. In general, how *comfortable* or *uncomfortable* do you feel about *your* health information being shared?

Very Uncom-fortable									Very Comfor-table
1	2	3	4	5	6	7	8	9	10

    B. How much value do you think health information sharing has for society?

Not at all valuable									Very valuable
1	2	3	4	5	6	7	8	9	10

The final questions are about your views of today's event.

Circle one answer:

21. Do you feel that your opinions were respected by your group?

Not at all									Very much
1	2	3	4	5	6	7	8	9	10

22. Do you feel you were listened to by your facilitator?

Not at all									Very much
1	2	3	4	5	6	7	8	9	10

23. Do you feel that the process that led to your group's responses was fair?

Not at all									Very much
1	2	3	4	5	6	7	8	9	10

24. How willing are you to abide by the group's final position, even if you personally have a different view?

Not at all									Very much
1	2	3	4	5	6	7	8	9	10

25. How helpful did you find each of the following?

a. *The formal presentations given by the experts.*

Not helpful at all									Extremely helpful
1	2	3	4	5	6	7	8	9	10

b. *Question and answer with experts.*

Not helpful at all									Extremely helpful
1	2	3	4	5	6	7	8	9	10

c. *Discussing the issues with other participants.*

Not helpful at all									Extremely helpful
1	2	3	4	5	6	7	8	9	10

26. How much did attending the session change your *understanding* of health information sharing?

Not at all									Very much
1	2	3	4	5	6	7	8	9	10

27. How much did attending the session change your *opinion* about health information sharing?

Not at all									Very much
1	2	3	4	5	6	7	8	9	10

28. Overall, how would you rate the quality of the deliberative session today?

Very low									Very high
1	2	3	4	5	6	7	8	9	10

29. Do you have any comments you wish to share about the session day or this study, in general?

Thank you for completing this survey!
